# Genomewide Association Study of African Children Identifies Association of *SCHIP1* and *PDE8A* with Facial Size and Shape

**DOI:** 10.1371/journal.pgen.1006174

**Published:** 2016-08-25

**Authors:** Joanne B. Cole, Mange Manyama, Emmanuel Kimwaga, Joshua Mathayo, Jacinda R. Larson, Denise K. Liberton, Ken Lukowiak, Tracey M. Ferrara, Sheri L. Riccardi, Mao Li, Washington Mio, Michaela Prochazkova, Trevor Williams, Hong Li, Kenneth L. Jones, Ophir D. Klein, Stephanie A. Santorico, Benedikt Hallgrimsson, Richard A. Spritz

**Affiliations:** 1 Human Medical Genetics and Genomics Program, University of Colorado School of Medicine, Aurora, Colorado, United States of America; 2 Department of Anatomy, Catholic University of Health and Allied Sciences, Mwanza, Tanzania; 3 Department of Anatomy and Cell Biology and McCaig Institute for Bone and Joint Health, University of Calgary, Calgary, Canada; 4 Hotchkiss Brain Institute, Cummings School of Medicine, University of Calgary, Calgary, Canada; 5 Department of Mathematics, Florida State University, Tallahassee, Florida, United States of America; 6 Laboratory of Transgenic Models of Diseases, Institute of Molecular Genetics of the ASCR, Prague, Czech Republic; 7 Department of Orofacial Sciences and Program in Craniofacial Biology, University of California San Francisco, San Francisco, California, United States of America; 8 Department of Craniofacial Biology, University of Colorado School of Dental Medicine, Aurora, Colorado, United States of America; 9 Department of Biochemistry and Molecular Genetics, University of Colorado School of Medicine, Aurora, Colorado, United States of America; 10 Department of Mathematical and Statistical Science, University of Colorado Denver, Denver, Colorado, United States of America; 11 Department of Biostatistics & Informatics, Colorado School of Public Health, Aurora, Colorado, United States of America; 12 Department of Pediatrics, University of Colorado School of Medicine, Aurora, Colorado, United States of America; Stanford University School of Medicine, UNITED STATES

## Abstract

The human face is a complex assemblage of highly variable yet clearly heritable anatomic structures that together make each of us unique, distinguishable, and recognizable. Relatively little is known about the genetic underpinnings of normal human facial variation. To address this, we carried out a large genomewide association study and two independent replication studies of Bantu African children and adolescents from Mwanza, Tanzania, a region that is both genetically and environmentally relatively homogeneous. We tested for genetic association of facial shape and size phenotypes derived from 3D imaging and automated landmarking of standard facial morphometric points. SNPs within genes *SCHIP1* and *PDE8A* were associated with measures of facial size in both the GWAS and replication cohorts and passed a stringent genomewide significance threshold adjusted for multiple testing of 34 correlated traits. For both *SCHIP1* and *PDE8A*, we demonstrated clear expression in the developing mouse face by both whole-mount *in situ* hybridization and RNA-seq, supporting their involvement in facial morphogenesis. Ten additional loci demonstrated suggestive association with various measures of facial shape. Our findings, which differ from those in previous studies of European-derived whites, augment understanding of the genetic basis of normal facial development, and provide insights relevant to both human disease and forensics.

## Author Summary

The human face is made up of distinct yet related anatomic structures that together make both individuals and families recognizable. It is clear there is a strong genetic component to the human face, and though the genetics of the face have been studied for several years, there are relatively few genes known to impact normal human facial development and facial shape. We report here a large-scale human genetic study in which we successfully identify and replicate genetic markers associated with normal facial variation using advanced 3D facial imaging in African children. We identified two significant replicated genes associated with measures of human facial size, *SCHIP1* and *PDE8A*, demonstrated their clear expression in the developing face in the mouse, and identified 10 additional candidate genetic loci for human facial shape. Gene discovery for human facial development is an important first step for both diagnosing and treating craniofacial syndromes and for developing forensic modeling of the human face.

## Introduction

The human face exhibits remarkable phenotypic variation, making it one of the most recognizable human characteristics. While facial variation is subject to environmental modifiers such as age and nutritional status, striking facial similarities within families suggest a strong genetic component [1], and heritability of some facial measurements is as high as 94% [2–6].

Nevertheless, candidate gene [1, 7–9] and genomewide association [10–12] studies of facial variation principally in adults have yielded few consistent results, possibly due to use of inconsistent phenotyping methods as well as confounding environmental differences such as nutritional status and age. A candidate gene study of various facial morphologic measures in a multiethnic cohort reported marginal association of SNPs in 20 genes, including *SLC35D1*, *FGFR1*, and *LRP6*. Genomewide association studies (GWAS) in European-derived white (EUR) adolescents [10, 11] and adults [11, 12] have reported associations of various midfacial phenotypes with SNPs in the *PAX3*, *TP63*, *COL17A1*, *C5orf50*, *PRDM16*, *DCHS2*, *RUNX2*, *GLI3*, *PAX1*, and *EDAR* regions.

Here, we describe a GWAS and two independent replication studies of facial size and shape measures in African Bantu children, a relatively homogeneous population in whom remarkably lean body mass and young age may minimize confounding environmental influences. We identified significant association of two loci with measures of facial size, and suggestive association of 10 additional loci with measures of facial shape. These results from the first GWAS of facial morphology reported for an African population, differ from those reported for EUR populations, and have potential implications for understanding facial development and facial birth defects, as well as forensic applications in modeling the human face from DNA.

## Results

**Fig 1:**
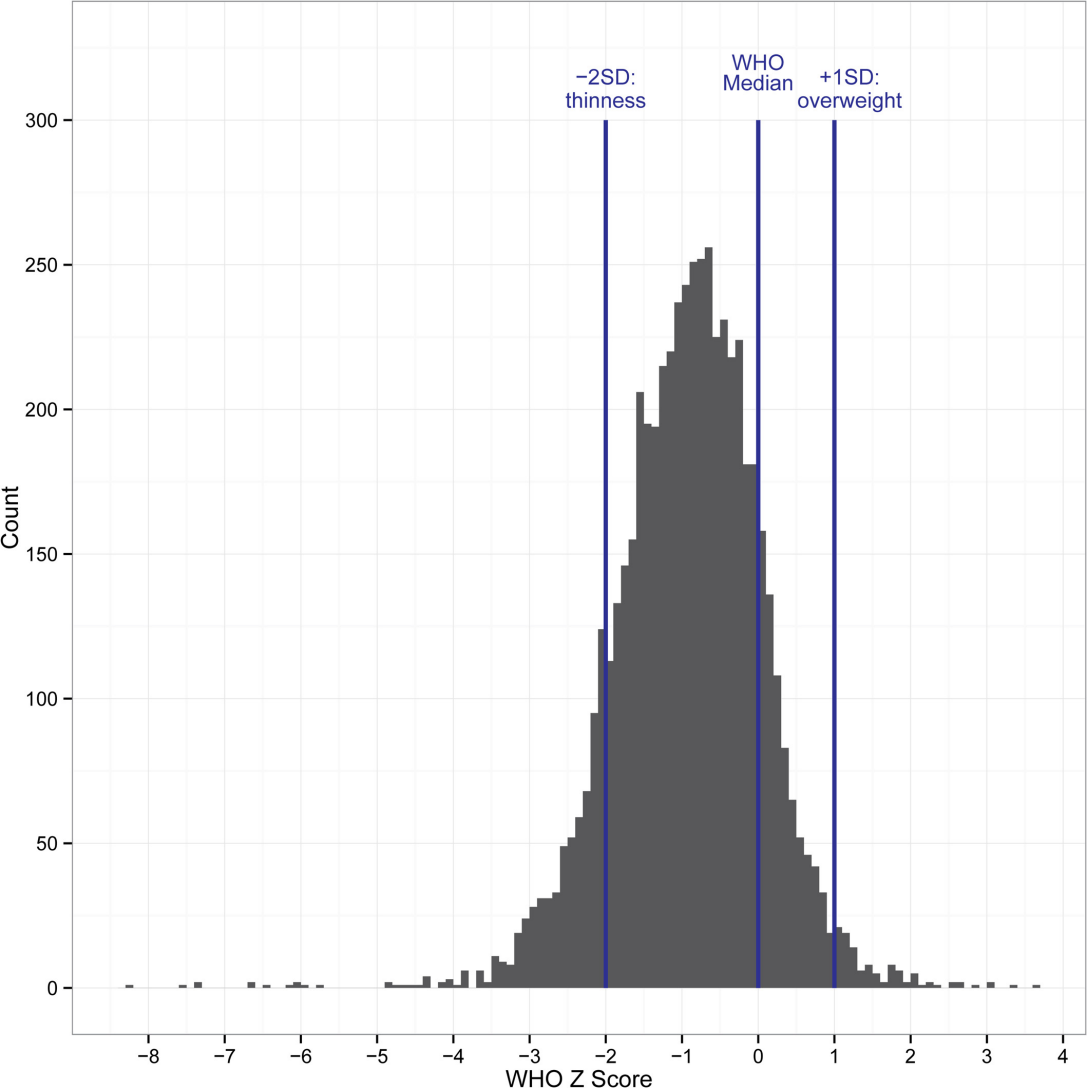
World Health Organization 2007 reference BMI Z-scores for the Tanzanian study population. Histogram of Tanzanian Z-scores calculated from WHO 2007 reference data by age and sex with WHO cutoffs for thinness and overweight classifications.

The GWAS cohort included 3,505 normal African Bantu children and adolescents ages 3–21 from the Mwanza region of Tanzania. Over 70% of subjects were aged 7 through 12 (median age = 10 years; interquartile range (IQR) = 8 to 13 years), and 45% were male and 55% female (**S1 Fig**). This study population was selected to minimize non-genetic confounders of facial morphology; namely, age and excess subcutaneous fat. Indeed, for both the GWAS and replication cohorts (see below), BMI is considerably lower than the WHO 2007 Reference; between the ages of 5–19 approximately 98% of our study population is below the WHO overweight classification (+1SD), 86% is below the WHO median, and 13% is below the WHO thinness classification (-2SD) (**Fig 1**). Furthermore, Mwanza is located approximately two degrees south of the Equator, and has a remarkably constant climate, minimizing environmental climatic confounders. Detailed analyses of potential population substructure in the GWAS cohort demonstrated the absence of apparent genetic subgroups by both school and tribe (**S2 Fig and S3 Fig**), with median fixation index (F_ST_) values of 0.0005 (IQR = 0.0002 to 0.0010) and 0.0020 (IQR = 0.0007 to 0.0043) for schools and tribes, respectively. Thus, this region is both environmentally and genetically relatively homogeneous.

The two independent replication cohorts (termed MC and GM) respectively consisted of 1,140 and 1,250 African Bantu children from the same population as the GWAS. Both replication cohorts had similar distributions of age and sex as the GWAS screening population; 66% of the MC replication cohort were aged 7 through 12 (median age = 11 years; IQR = 9 to 13 years); 45% were male and 55% were female (**S1 Fig**). Similarly, 77% percent of the GM replication cohort were aged 7 through 12 (median age = 11 years; IQR = 9 to 12 years); 41% were male and 59% were female (**S1 Fig**). Our GWAS and two replication cohorts have no known or apparent differences in age range, sex representation, tribal composition, or representation of locales. The GWAS cohort and MC replication cohort were both imaged using the MC 3D camera system. After failure of the MC camera in the field, the GM replication cohort was imaged using the very similar GM 3D camera system. Although the MC and GM camera systems yielded very similar data, to minimize potential bias the MC and GM replication cohorts were analyzed separately, and the test statistics combined by meta-analysis.

**Fig 2:**
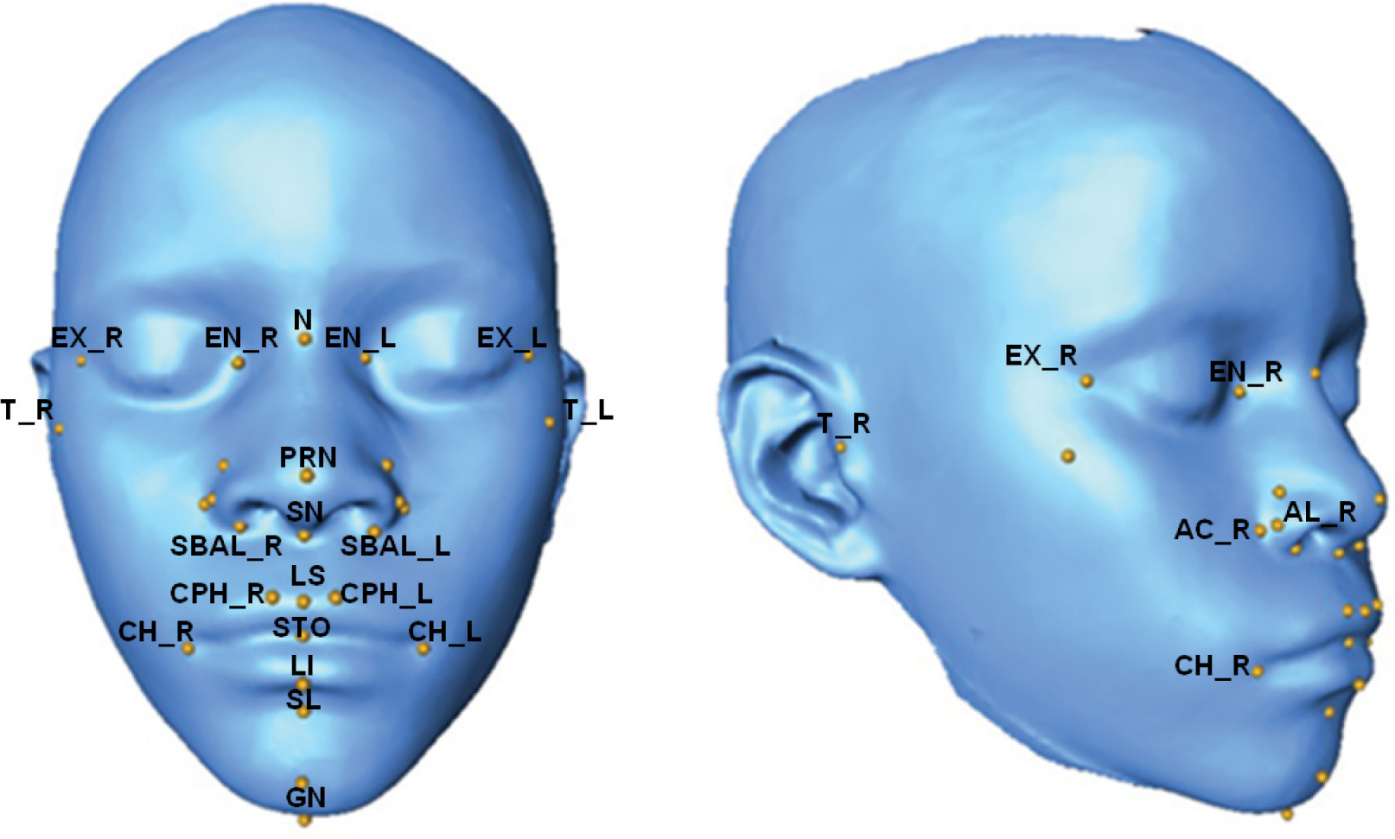
3D Facial scan with annotated landmarks. 3D facial mesh obtained for each study individual with study landmarks obtained from automatic landmarking overlaid and labeled.

Quantitative phenotypes were derived from the MC and GM 3D facial scans based on 29 standard facial morphometric landmarks (**Fig 2**). These quantitative phenotypes were of three general types (**Table 1**), including three global measures of facial size, 25 inter-landmark linear distances, five summary variables from a principal components analysis (PCA) of the whole face (explaining approximately 70% of total facial variation) (**S4 Fig**), and one summary variable from a PCA of the most highly correlated mid-facial landmarks (explaining approximately 40% of total midface variation) (**S5 Fig**). We then used a specialized statistical approach for quantitative association testing in our GWAS cohort to account for occult relatedness in the sample (see Material and Methods). Following GWAS and replication study analyses, we performed a meta-analysis to combine statistics of the GWAS and two replication datasets.

**Table 1:** Facial size and shape phenotypes tested for genetic association.

Phenotype Abbreviation	Physical Description
**Size-related Measurements**	
Centroid Size	facial size
Allometry	variation in shape due to size
Head Circumference	direct occipitofrontal circumference
**Linear Distances**	
AL_R_AL_L	nasal width
AC_PRN	nasal ala length (average)
CH_R_CH_L	mouth width
CPH_R_CPH_L	philtrum width
EN_EX	palpebral fissure length (average)
EN_R_EN_L	inner canthal width
EX_R_EX_L	outer canthal width
GN_T	lower facial depth (average)
LI_SL	cutaneous lower lip height
LS_STO	upper vermilion height
N_GN	morphological facial height
N_MEN	nasion to midendocanthion
N_PRN	nasal bridge length
N_SN	nasal height
N_STO	upper facial height
N_T	upper facial depth (average)
SBAL_R_SBAL_L	subnasal width
SN_GN	lower facial height
SN_LS	philtrum length
SN_PRN	nasal protrusion
SN_STO	upper lip height
SN_T	midfacial depth (average)
STO_LI	lower vermilion height
STO_SL	lower lip height
T_R_T_L	facial width
**Principal Components Analysis (PCA)**	
Principal Component 1 (PC1)	upper facial height, midfacial width
Principal Component 2 (PC2)	overall facial shape, upper facial width
Principal Component 3 (PC3)	upper facial depth, prognathism
Principal Component 4 (PC4)	facial height, nasal width
Principal Component 5 (PC5)	midface width and projection
PC1 from a PCA of the mid-facial landmark network (MidfaceModPC1)	midfacial landmark network around the nose and mouth

Linear distances are the distances between two landmarks (e.g. AL_R and AL_L).

**Fig 3:**
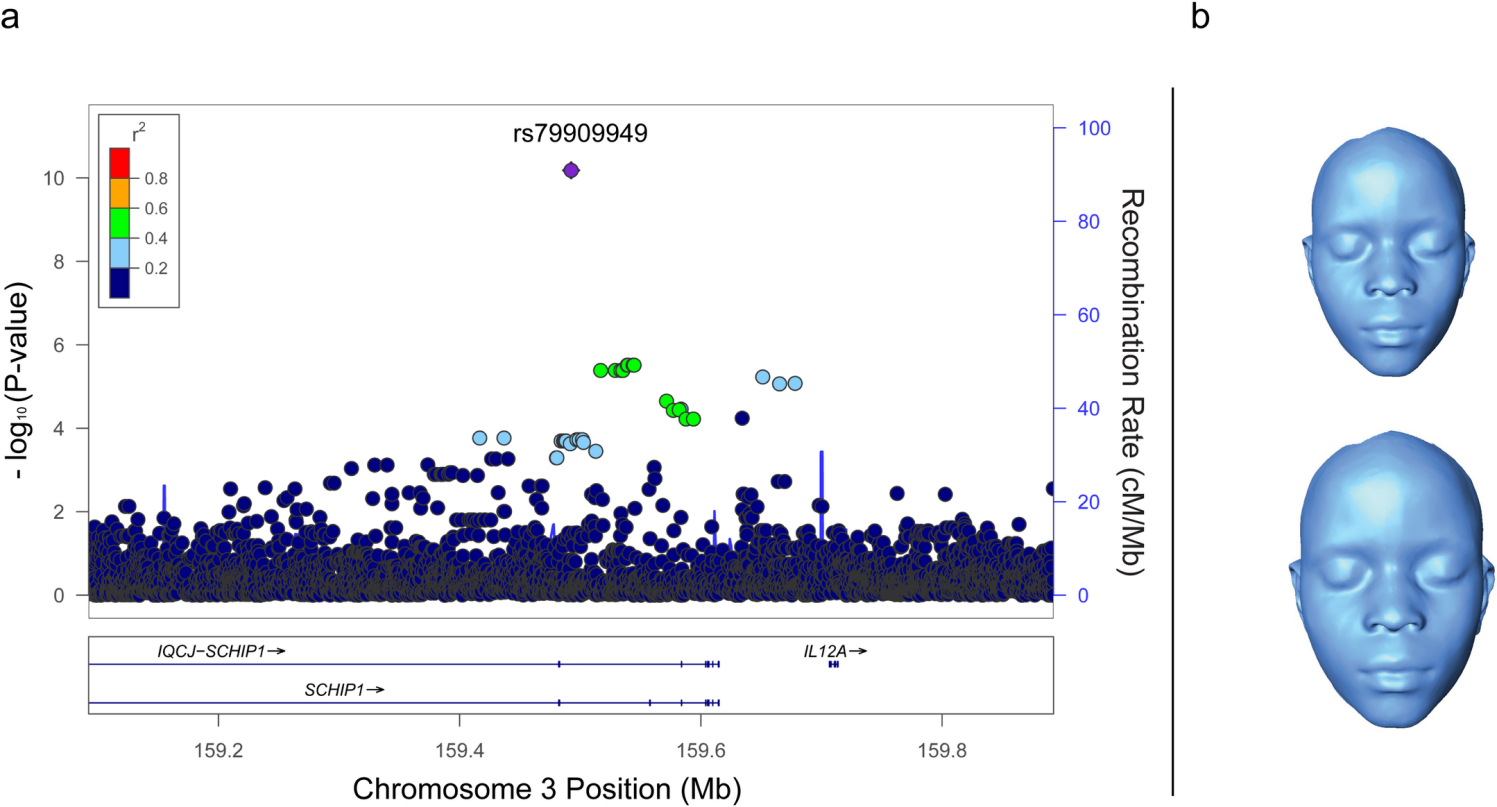
*SCHIP1* locus associated with centroid size. **(A)** Regional association plot of centroid size at the *SCHIP1* locus. Association data are shown using GWAS P-values with the meta-analysis P-value for the lead SNP, rs79909949. The LD pattern is based on the 1000 Genomes Project 2012 African reference and GRCh37/hg19. The estimated recombination rate (cM/Mb) is from HapMap samples. **(B)** Relative facial size at the upper and lower 95% confidence intervals for centroid size after adjusting for sex and age.

Two loci were associated with distinct measures of global facial size, both associations surpassing a genomewide significance criterion for an African population (P < 2.50 x 10^−8^) [13], yielding independent replication (P < 0.05) with no significant inter-study heterogeneity (I^2^ < 50%), and surpassing a stringent genomewide meta-analysis significance threshold corrected for number of effectively independent phenotypes tested (P < 2.50 x 10^−8^ / 9 = 2.78 x 10^−9^). The first of these associations was of SNPs in the *SCHIP1* region of chromosome 3q25.33 (chr3:159,774,689–159,960,389; **Fig 3A** and **S6 Fig**) with centroid size, a measure of overall facial size that is uncorrelated with variables of shape [14] (**Fig 3B**). Twenty SNPs in the *SCHIP1* region were associated with centroid size in the GWAS. The lead SNP was rs79909949 (GWAS P = 9.56 x 10^−9^; replication P = 1.80 x 10^−3^; meta-analysis P = 6.58 x 10^−11^), located within *SCHIP1*, 500 kb downstream of the 5’ transcriptional start for *SCHIP1* transcripts 1, 2, and 3 and 65 kb upstream of the unique 5’ transcriptional start for *SCHIP1* transcript 4, which contains an alternative first exon. SNP rs79909949 is within a striking ENCODE [15] predicted transcriptional regulatory element (chr3:159,491,500–159,495,250) that, as predicted by HaploReg v4.1 (http://www.broadinstitute.org/mammals/haploreg/haploreg.php), exhibits features of an enhancer in many different cell types including an open hypomethylated chromatin configuration, multiple DNase I hypersensitivity sites, and numerous RNA polymerase II and transcription factor binding sites. HaploReg v4.1 predicts that SNP rs79909949 alter bindings motifs of at least three different transcription factors.

**Fig 4:**
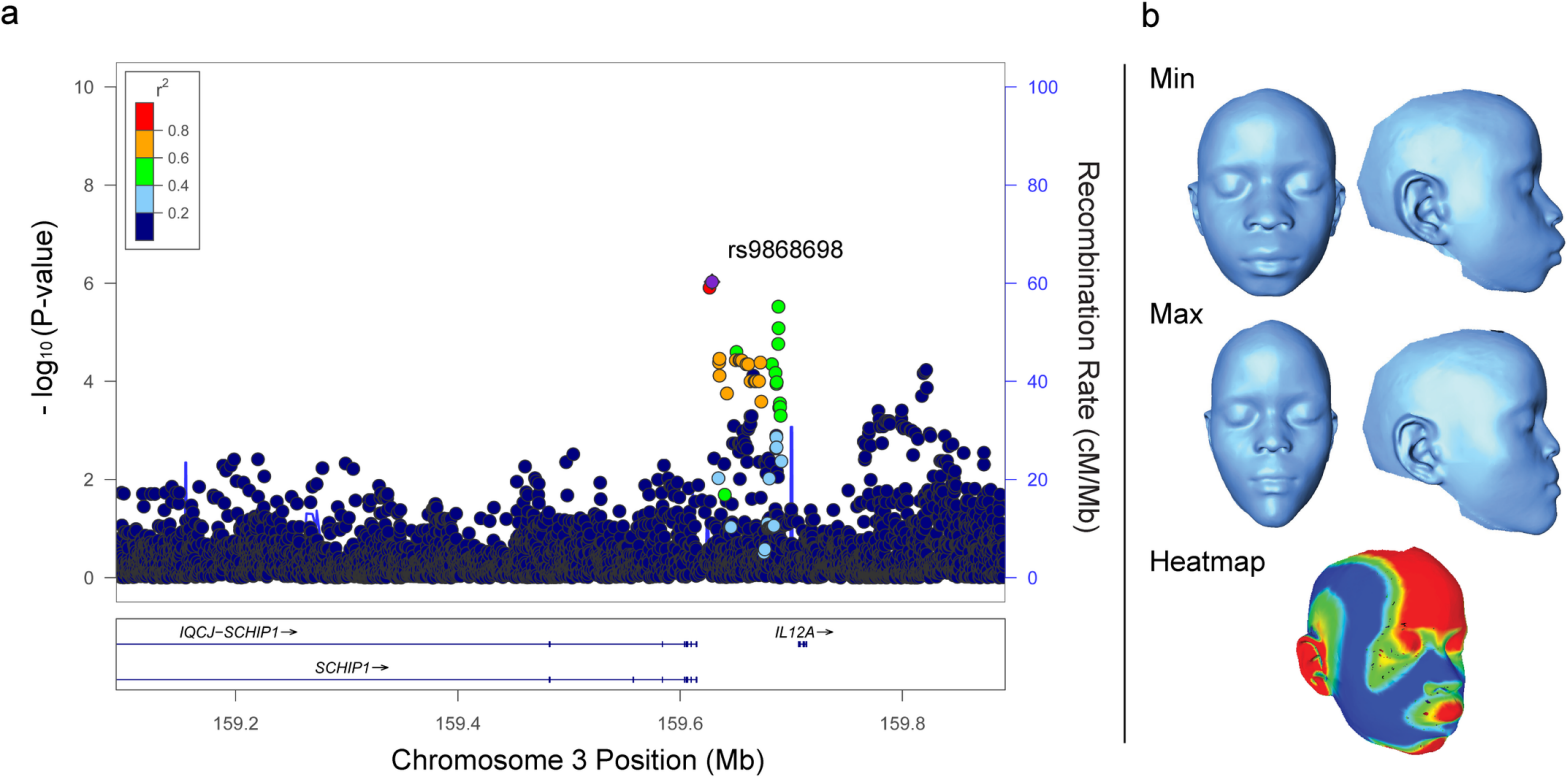
*SCHIP1* locus associated with PC4. **(A)** Regional association plot of PC4 at the *SCHIP1* locus. Association data are shown using GWAS P-values. The most associated SNP rs368386044 could not be displayed in the LocusZoom plot, but is in complete linkage disequilibrium with rs9868698. See **Fig 3**legend for details. **(B)** Morphs showing the range of shape variation along PC4. The heatmap depicts the regions of the face that vary the most between the min and max morphs. Red shows the regions that project most beyond the mean mesh at the positive extreme while yellow is intermediate in that direction. Blue shows the areas that project most inwards from the mean mesh while light blue shows a lesser degree of inwards projection. Green shows those regions that align most closely to the mean mesh.

Furthermore, 35 additional SNPs in a largely overlapping region within *SCHIP1* (chr3:159,908,007–160,103,634; **Fig 4A** and **S6 Fig**) were associated with PC4, representing both facial height and nasal width (**Fig 4B**), with P-values as low as 7.92 x 10^−7^ (rs368386044). Conditional analysis showed that the PC4 association was independent of the centroid size association; conditioning the lead PC4 SNP rs368386044 on the lead centroid size SNP rs79909949 did not affect significance (after conditioning P = 7.95 x 10^−7^). Thus, rs79909949 is associated with overall facial size while rs63740860 is independently associated with facial height and nasal width.

**Fig 5:**
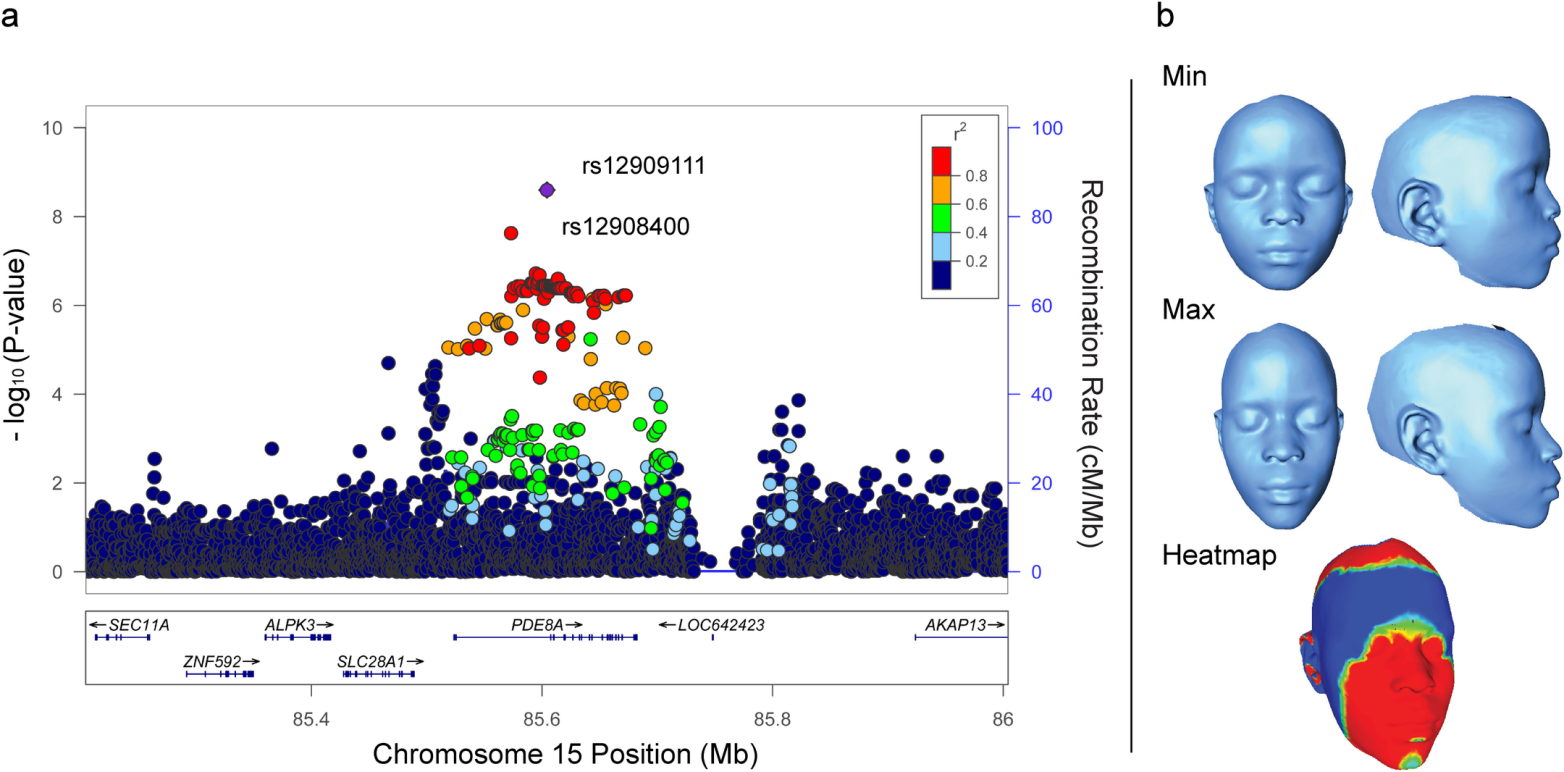
*PDE8A* locus associated with allometry. **(A)** Regional association plot of the allometry variable at the *PDE8A* locus. Association data are shown using GWAS P-values with the meta-analysis P-values for the two lead SNPs, rs12909111 and rs12908400. See **Fig 3**legend for details. **(B)** Morphs showing the range of allometric variation in facial shape. See **Fig 4**legend for heatmap details.

The second association was of SNPs in the *PDE8A* region of chromosome 15q25.3 (chr15: 84,923,649–85,161,983; **Fig 5A** and **S6 Fig**) with the allometry variable, which represents a complex scaling relationship between size and shape (**Fig 5B**). One hundred thirty-six SNPs in the *PDE8A* region were associated with the allometry variable in the GWAS. The lead SNP, rs12909111 (GWAS P = 2.36 x 10^−7^; replication P = 1.53 x 10^−3^; meta-analysis P = 2.52 x 10^−9^), is located within intron 1 of *PDE8A* transcripts 2, 3, and 4 and intron 2 of *PDE8A* transcript 1. We also replicated association of a second SNP within *PDE8A*, rs12908400 (GWAS P = 1.92 x 10^−7^; replication P = 1.03 x 10^−3^; meta-analysis P = 2.36 x 10^−8^), in strong linkage disequilibrium (D' = .91, r^2^ = .81) with rs12909111. SNP rs12908400 is located 31 kb upstream of rs12909111, within a broad ENCODE predicted transcriptional element observed in endothelial cells, and HaploReg v4.1 predicts that rs12908400 overlaps an enhancer active in many different tissue types, alters 10 transcription factor binding motifs, and overlaps 4 apparent eQTL tissue associations.

Furthermore, 49 of the *PDE8A* region SNPs associated with the allometry variable also showed marginal association with other facial phenotypes. Forty-one of these SNPs were associated with inner canthal distance EN_R_EN_L, and eight were associated with PC2, representing overall facial shape and upper facial width. Conditional analysis showed that the EN_R_EN_L association was completely dependent on the allometry association; conditioning on the lead allometry SNP rs12909111 abolished significance for the lead EN_R_EN_L SNP rs57482637 (before conditioning P = 2.60 x 10^−5^; after conditioning P = .059). However, conditional analysis showed that the PC2 association was partially independent of allometry; conditioning on the lead allometry SNP rs12909111 did not abolish significance for the lead PC2 SNP rs141832194 (before conditioning P = 8.75 x 10^−6^; after conditioning P = 3.42 x 10^−4^). Thus, rs12909111 is associated with the allometry facial size and shape variable while rs63740860 may be independently associated with overall facial shape and upper facial width.

In addition to these two confirmed associations, 11 additional SNPs in ten loci showed suggestive association, defined by nominal GWAS association (P < 1.0 x 10^−5^), nominal replication (P < 0.05), marginal meta-analysis significance (P < 1.0 x 10^−6^), and no significant inter-study heterogeneity (I^2^ < 50%) (**Table 2**). Two different SNPs in complete linkage disequilibrium at the *TFAP2B* locus at 6p12.3 were associated with two different highly correlated eye measurement phenotypes (r^2^ = .75), EX_R_EX_L, representing outer canthal width, and EN_EX, representing average palpebral fissure length. SNP rs2817419, within the *TFAP2B* 3' untranslated region (UTR) was associated with both outer canthal width (meta-analysis P = 7.28 x 10^−8^) and average palpebral fissure length (GWAS P = 5.69 x 10^−6^). SNP rs35965172, located 2 kb downstream of the *TFAP2B* 3' UTR was associated with average palpebral fissure length (meta-analysis P = 4.82 x 10^−7^) and outer canthal width (GWAS P = 9.41 x 10^−6^). Conditional analysis demonstrated complete dependence of these two SNPs with outer canthal width and average palpebral fissure length; conditioning on the lead SNP rs2817419 abolished significance for SNP rs35965172 with average palpebral fissure length (before conditioning GWAS P = 3.57 x 10^−6^; after conditioning P = .1574). *TFAP2B* encodes AP-2ß, a key transcription factor for craniofacial development, and heterozygous missense mutations in *TFAP2B* cause Char Syndrome, which includes dysmorphic facial features such as hypertelorism, downward slanting palpebral fissures, flattened and broad nose, short philtrum, and triangular mouth with prominent upper lips [16, 17]. *TFAP2B* is thus a strong biological candidate for involvement in facial shape variation, particularly involving the region around the eyes.

**Table 2:** GWAS, replication, and meta-analysis results of replicated association signals.

SNP	Trait	CHR	NT	Gene	Effect Allele	GWAS MAF	GWAS P-value	GWAS *β*	GWAS SE^c^	Replication MAF^a^	Replication 1-sided P-value^a^^,^^b^	Meta-analysis I^2^ heterogeneity	Meta-analysis P-value^b^	Meta-analysis *β*	Meta-analysis SE^c^
rs79909949	Centroid Size	3	159774689	*SCHIP1*	C	0.021	9.56E-09	-4.32	0.75	0.0044	1.80E-03	0.0	6.58E-11	-4.47	0.70
rs12909111	Allometry	15	85061095	*PDE8A*	G	0.30	2.36E-07	0.0027	0.00052	0.31	1.53E-03	12.0	2.52E-09	0.0023	0.00040
rs12908400	Allometry	15	85029945	*PDE8A*	G	0.30	1.92E-07	0.0027	0.00051	0.30	1.03E-02	35.6	2.36E-08	0.0021	0.00040
rs7836044	STO_SL	8	136880907	intergenic	T	0.47	5.69E-08	-0.17	0.032	0.50	3.08E-02	46.1	2.70E-08	0.14	0.025
rs139879053	PC1	1	23313306	*HNRNPR*	T	0.024	3.63E-07	-0.0084	0.0016	0.012	2.04E-02	8.0	2.73E-08	-0.0078	0.0014
rs148037459	EX_R_EX_L	9	93212785	*WNK2*	A	0.086	4.32E-06	-0.42	0.091	0.092	3.58E-03	0.0	5.88E-08	-0.39	0.073
rs2817419	EX_R_EX_L	6	50845193	*TFAP2B*	G	0.37	4.75E-07	-0.27	0.053	0.36	2.18E-02	0.5	7.28E-08	-0.23	0.043
rs35965172	EN_EX	6	50849828	2 kb downstream *TFAP2B*	T	0.31	3.57E-06	-0.11	0.025	0.32	2.39E-02	0.0	4.82E-07	-0.097	0.020
rs114189713	CH_R_CH_L	7	154532074	*DPP6*	A	0.039	1.84E-07	-0.62	0.12	0.037	2.64E-02	45.7	8.35E-08	-0.47	0.090
rs7627283	PC2	3	16192201	*GALNT15*	A	0.39	1.02E-06	0.0023	0.00048	0.37	2.91E-02	45.0	4.01E-07	0.0018	0.00040
rs75004472	LI_SL	15	27252846	*GABRG3*	G	0.14	3.25E-06	0.18	0.041	0.15	2.40E-02	32.0	7.47E-07	0.15	0.031
rs12112855	PC3	7	155736005	*RBM33*	T	0.076	1.55E-06	-0.0040	0.00082	0.071	4.08E-02	37.5	8.08E-07	-0.0030	0.00060
rs73936436	SBAL_R_SBAL_L	2	65216165	10kb upstream *ACTR2*	T	0.036	6.02E-06	-0.43	0.094	0.039	1.50E-02	24.5	9.74E-07	-0.32	0.067
rs61448485	N_MEN	2	72243866	*EXOC6B*	A	0.36	3.82E-06	-0.14	0.031	0.37	3.66E-02	13.5	9.92E-07	-0.12	0.025

^a^Replication MAF and P-value are combined from the meta-analysis of the two individual replication studies by camera

^b^Replication and meta-analysis P-values are 1-sided

^c^SE, standard error of *ß*

**Fig 6:**
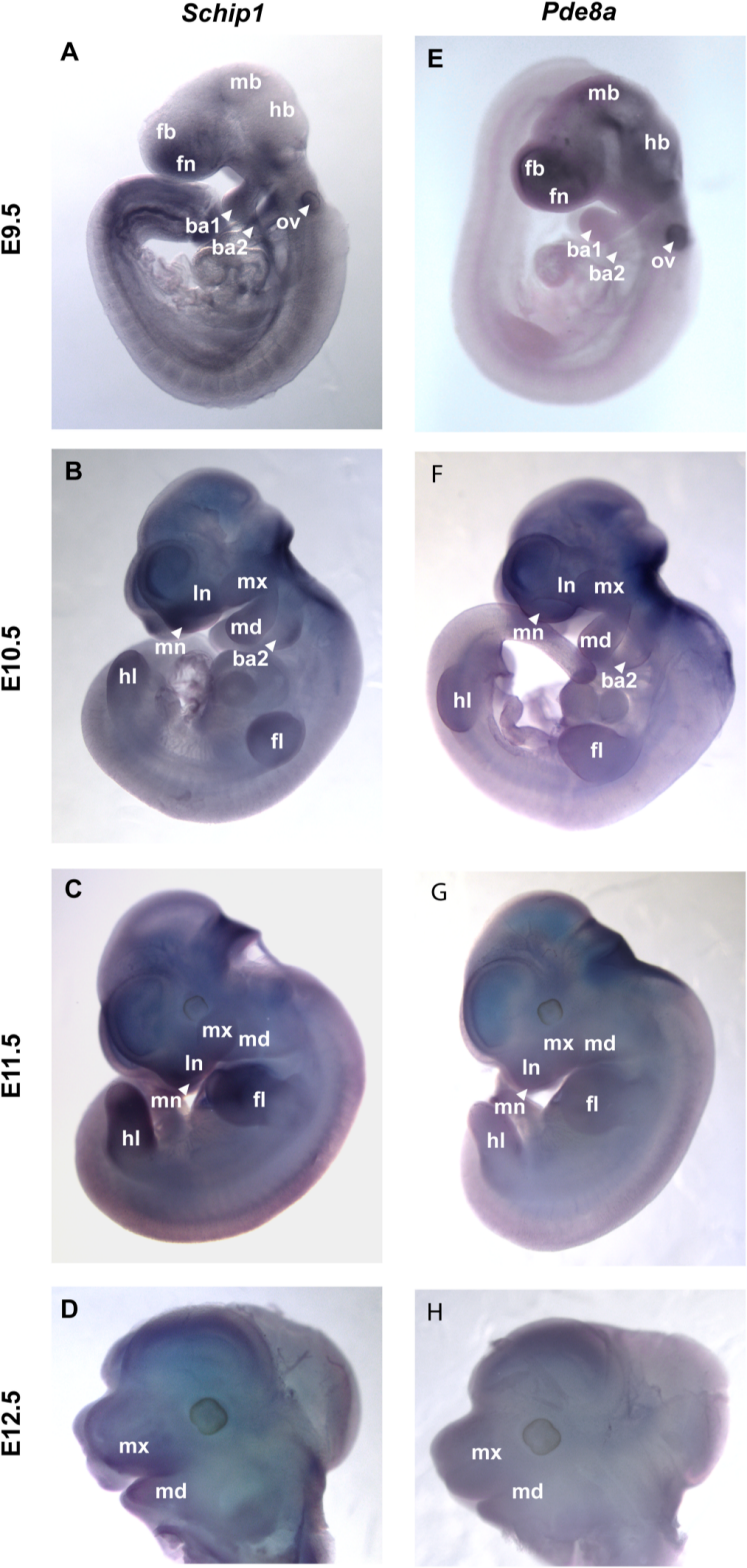
Expression of *Schip1* and *Pde8a* during mouse embryonic development. Whole-mount *in situ* hybridization of **(A-D)** *Schip1* and **(E-H)** *Pde8a* expression in mouse embryos from E9.5 to E12.5. ba1, first branchial arch (future mandible); ba2, second branchial arch; fb, forebrain; fn, frontonasal process; fl, forelimb; hb, hindbrain; hl, hindlimb; ln, lateronasal process; mb, midbrain; md, mandible; mn, medionasal process; mx, maxilla; ov, otic vesicle.

*SCHIP1* and *PDE8A* have not previously been implicated in facial morphogenesis. To assess their roles, we assayed expression of *Schip1* and *Pde8a* during mouse development. Quantitation of gene expression in microdissected murine facial tissues at E9.5-E12.5, a critical period of mouse facial development, by both expression microarray [18] and RNAseq analyses showed that both *Schip1* and *Pde8a* are differentially expressed in the developing mouse face. As shown in **Fig 6**, in whole-mount embryos *Schip1* is expressed in multiple tissues, including the developing face; specifically, the nasal processes, maxillary process, and mandibular process. Expression appears maximal at E10.5 and 11.5, and then declines (**Fig 6A–6D**). In addition, RNAseq analysis of *Schip1* demonstrated high expression in the ectoderm of the nasal, maxilla, and mandibular prominences at E11.5 and E12.5, with somewhat less expression in the mesenchyme (**S7 Fig**). Similarly, in whole-mount embryos *Pde8a* was expressed principally in the face, maximal at E9.5 (**Fig 6E–6H**). RNAseq analysis of *Pde8a* demonstrated that expression occurs primarily in the mesenchyme of all facial prominences, with little, if any, ectodermal expression during these same periods of critical development (**S7 Fig**).

## Discussion

The genetic underpinnings of facial shape is a topic of considerable scientific, forensic, and popular interest, and familial aspects of a newborn’s face are one of the first characteristics noted by grandparents around the world. The strong facial resemblance among close relatives, and the high heritabilities of many facial measurements [2–6], underscore a major genetic component underlying facial shape. It is clear that aging and environmental factors such as nutrition also play large roles in facial shape. Nevertheless, the intriguing ability of so-called “super-recognizers” to identify individual faces regardless of environmental variables [19] suggests that each human face has its own intrinsic shape, remarkably constant and almost certainly genetically determined. The amazing ability of *Polistes fuscatus* paper wasps to similarly use facial recognition and learning to distinguish between friend and foe [20] indicates a remarkable phenomenon that transcends beyond the evolution of the human species.

Our findings demonstrate that both *SCHIP1* and *PDE8A* are associated with measures of human facial size, and both are expressed in the developing face in the mouse. SCHIP1 was first identified as a major protein that interacts with schwannomin, the neurofibromatosis type 2 tumor suppressor protein [21]. Initial characterization of *SCHIP1* cDNA and mRNA indicated that it is a coiled-coil protein expressed principally in the brain, skeletal muscles, and heart and to a lesser extent in the pancreas, kidney, liver, lung, and placenta; however, bone, neural crest derivatives, and other tissues relevant to the face were not analyzed [21]. Subsequent studies showed that *Schip1* knockout mice exhibit developmental anomalies of the neural-crest derived skeleton, the palatal processes, and the snout [22], indicating that it also plays a role in facial development. SCHIP1 functions as an early response gene in the PDGF signaling cascade, promoting cell migration in response to PDGF signaling through actin cytoskeleton rearrangements [23], though its specific function in the developing face remains unknown.

PDE8A was first identified as a novel cyclic nucleotide phosphodiesterase via a bioinformatic screen based on PDE family homology [24]. This study demonstrated predominant expression in the testis and ovary, with minimal expression in several other tissues, but again, did not assess expression in bone or other relevant craniofacial tissues [24]. PDE8A hydrolyzes cAMP, regulates Ca^2+^ movement through cardiomyocytes [25], and stimulates testosterone production in Leydig cells [26]. The function of PDE8A in the developing face is not known.

This is one of the first studies to demonstrate replicated genomewide genetic associations of human facial morphometric phenotypes in humans, and is the first reported in an African population. We did not replicate previous reported facial shape associations [10–12], from studies of EUR populations. It is possible that facial morphology differences in different human populations have different genetic underpinnings. Alternatively, as noted above, our study cohort was young and almost universally lean, and therefore may be less influenced by environmental factors than study cohorts of adults from EUR populations. Aside from an interesting overlap in *TFAP2B* which is known to cause Char syndrome, the majority of our newly identified genetic loci have not been previously implicated in human facial development, facial dysmorphic syndromes, or animal mutant models. Our findings provide a basis for detailed analyses of the functions of these genes in the developing face, and their roles in determining the normal facial variation that make us both individually different and individually recognizable.

## Materials and Methods

### Study Subjects

Sample and data collection for both the GWAS screening cohort and two replication cohorts of Bantu African children was undertaken under the NIDCR FaceBase1 initiative, over a three-year period in the Mwanza region of Tanzania. The GWAS screening cohort included 3,631 subjects, all imaged using the Creaform MegaCapturor (MC) camera three-dimensional (3D) photogrammetry imaging system. The first replication cohort included 1,173 subjects also imaged using the MC 3D system, and the second replication cohort included 1,506 subjects imaged using the Creaform Gemini (GM) 3D imaging system. All subjects were apparently unrelated, aged 3–21. Individuals with a known birth defect or a relative with a known orofacial cleft were excluded. Additional data collected from each subject included age, sex, height, weight, school, and detailed parental and grandparental ethnicity and tribe information. Tanzanian BMI Z-scores were calculated from the 2007 World Health Organization (WHO) growth reference 5–19 year old distribution [27] for all individuals with non-missing height and weight. Written informed consent was obtained for all study subjects or their parents, as appropriate. This study was carried out with overall approval and oversight of the Colorado Multiple Institutional Review Board (protocol #09–0731), was additionally approved by the institutional review boards of the University of Calgary, Florida State University, the University of California San Francisco, and the Catholic University of Health and Allied Sciences (Mwanza, Tanzania), and was carried out with the approval of the National Institute for Medical Research (Tanzania). Written informed consent was obtained from all study subjects or their parents, as appropriate.

### Derivation of Phenotypic Variables

Each subject was imaged twice at six angles. Individual images were assembled at the highest possible resolution into a single 3D mesh composite of the face using InSpeck FAPS and EM 6.0 software. Twenty-nine 3D landmarks were placed on the meshes using a novel automated landmarking method. A manuscript describing the automated landmarking method has been submitted for publication. Details of the method and the automated landmarking algorithm are available at https://www.facebase.org/facial_landmarking/ [28], and landmarks were then subjected to Procrustes superimposition for morphometric analysis [28–30]. Images from 163 subjects in the GWAS cohort were landmarked manually as they could not be landmarked automatically. This was mostly due to imaging artifacts on non-critical regions of the face that do not interfere with manual landmark placement. Superficial artifacts (smiling, squinting, open mouth, etc.) in the landmark data were corrected using a multiple linear regression in which all factors and their interactions were considered. The resulting residuals were mean centered and used for downstream phenotype derivation.

A 3D "skew" artifact (coordinated asymmetric displacement of landmarks due to image assembly) was identified by principal components analysis (PCA) of the landmark coordinates and was removed by regressing out the PC scores from the landmark data. Unlike manual landmarking, automated landmarking produces a non-normal distribution of measurement error; therefore, outliers were detected using the combination of Procrustes distance from the mean and the within-landmark variance of distances from each landmark mean. The cleaned landmark data were then used to calculate linear distances and multivariate measures to be used as phenotypes. Linear distance phenotypes were calculated as the distance between their defining landmarks, multiplied by the centroid size of each individual's landmark configuration. Centroid size, by definition, is the mean squared distance of each landmark from the geometric center. Allometry is shape variation related to size [31,32], and was calculated using regression scores corresponding to size independent of age. To calculate multivariate measures, we regressed out age and size variation in symmetrized landmark data, and the first five PCs of a PCA were used as phenotypes. **S1 File** shows 3D faces that correspond to the extremes of variation in allometry and the first 5PCs. To calculate the MidfaceModPC1 phenotype we used the RV method to identify the set of spatially contiguous landmarks that maximized the ratio of covariation among themselves to covariation with landmarks outside of that set [33]. The resulting set around the midface was then subjected to PCA, and the first PC represented MidfaceModPC1. For all variables, measurements greater than four standard deviations from the mean were excluded from analyses. All morphometric analyses were performed in MorphoJ [34] or in R using the Geomorph [35] and Morpho [36] packages. Head circumference was measured directly using a tape measure on a subset of 2,676 subjects. Phenotype data were deposited in the FaceBase data Hub (FaceBase: https://www.facebase.org/; FB00000667.01).

### GWAS Genotyping and QC

Subject saliva specimens were obtained using Oragene DNA self-collection kits (DNA Genotek), and genomic DNA was prepared from saliva specimens per the manufacturer's instructions or using the Maxwell™ robotic platform and Maxwell™ 16 Blood DNA Purification Kit (Promega). For genome-wide genotyping, DNA concentrations were assayed by fluorescence with the Qubit dsDNA BR Assay Kit (Life Technologies). Genome-wide genotyping was performed at the Center for Inherited Disease Research (CIDR) using the Illumina HumanOmni2.5Exome-8v1_A array, interrogating 2,567,845 variants. Genotypes were called using GenomeStudio ver. 2011.1, genotyping module 1.9.4 and GenTrain version 1.0. Quality control filtering of genome-wide genotype data was performed by the University of Washington Genetics Coordinating Center (UWGCC), as described elsewhere [37]. A total of 3,631 samples were genotyped successfully. Subjects were excluded on the basis of SNP call rates < 97% (*n =* 0), discordance between reported and genotyped sex (*n =* 0), XXY karyotypes (*n =* 2), non-Bantu heritage (*n =* 33), missing covariates (*n =* 2), and/or inadvertent subject duplication (*n =* 74). The final GWAS included 3,505 individuals after phenotype QC. Quality control analysis discovered considerable cryptic relatedness among our study population at the full or half-sibling level. This included 563 families of at least two members each, with all pairs of subjects connected by a kinship coefficient (KC) greater than the lower limit of the 95% prediction interval for half-siblings (coefficient > 0.098). As described below, we used a specialized statistical approach to account for this high level of relatedness in our association testing.

To more closely assess the genetic substructure within our population we performed a PCA on our unrelated cohort of 2,720 GWAS individuals, defined by KC less than the lower limit of the 95% prediction interval for half-siblings (KC < 0.098) using LD-pruned common markers genomewide, and color-coded PCs by both school and tribe (**S2 Fig**). Furthermore, we estimated F_ST_ between schools and tribes of all unrelated study subjects to determine if these defined subgroups identify genetically distinct sub-populations. We estimated F_ST_ within our unrelated cohort using LD-pruned common markers on chromosome 22. The estimates of F_ST_ by school were made using all unrelated individuals (*n* = 2,720) and the estimates of F_ST_ by tribe were made using all unrelated individuals with the same maternal and paternal tribe affiliations (*n* = 2,257). The distributions of pairwise F_ST_ estimates by school and tribe are depicted in **S3 Fig**. We limited this analysis to subgroups with *n* ≥ 5, as very small sample size has been shown to inflate F_ST_ estimates [38]. All pairwise estimates were < 0.05, supporting a high level of genetic homogeneity among our study subjects [39].

SNPs were excluded on the basis of call rates < 2% (*n =* 40,146), MAF < 1% (*n =* 602,288), positional duplicates (*n =* 39,847), CIDR technical filters (*n =* 58,825), > 1 discordant call in planned study duplicates (*n =* 900), > 1 Mendelian error in seven HapMap trios (*n =* 436), significant deviation (*P* < 10^−4^) from Hardy-Weinberg equilibrium (HWE; *n =* 7,856), and sex difference in allele frequency ≥ 0.2 (*n =* 199) leaving a total of 1,817,348 genotyped SNPs passing QC filters. The UWGCC performed genome-wide imputation of non-genotyped markers to 1000GenomesProject data using SHAPEIT2 and IMPUTE2 software for phasing and imputing probabilistic genotypes using a worldwide reference panel of all samples from The 1000 Genomes Project phase 1 integrated variant set [40]. Genotype data were deposited in the Database of Genotypes and Phenotypes (dbGaP: http://www.ncbi.nlm.nih.gov/gap; phs000622.v1.p1).

### Replication Genotyping and QC

DNA of the two replication study cohorts, collected as described above for the GWAS, was genotyped at CIDR using a custom 384 SNP Illumina GoldenGate assay per the manufacturer's instructions. From the two replication cohorts a total of 2,679 samples were genotyped successfully. Using the break in the individual genotype call rate distribution as a guideline, subjects with missing genotype rate > .04 (*n =* 7) or discordance between reported and observed sex (*n =* 12) were removed. Further individuals were removed due to poor image quality, duplicate collection, and landmarking outliers. The final replication dataset included 2,390 individuals. 231 SNPs plus five internal controls were genotyped successfully with call rate > 97.5%. SNPs that failed the Hardy-Weinberg equilibrium (HWE) test with a P-value Bonferroni-corrected for the number of independent signals to be tested (P < 2.52 x 10^−4^; 0.05/198) were excluded (*n =* 4), leaving a total of 227 SNPs that represented 194 independent loci.

### Statistical Analysis

We tested for association between each SNP and 34 phenotypic traits using the Efficient Mixed-Model Association eXpedited (EMMAX) method [41], implemented in the web-based BC Platforms data management software (http://bcplatforms.com/). A linear mixed-model with an additive SNP effect was used to account for both population stratification and a large amount of occult relatedness among our subjects. The specific phenotypes analyzed for genetic association included the first five PCs from a PCA of the whole face, the first PC from a PCA of the midface, 25 inter-landmark linear distances, centroid size, allometry, and head circumference (**Table 1**). All multivariate measures and linear distances were corrected for age, sex, and size at either the phenotype derivation or genetic association testing stages. In particular, multivariate measures, including all PCs and allometry, were adjusted for sex in the genetic model; centroid size was adjusted for age and sex; and linear distances were adjusted for age, sex, and centroid size (following adjustment for age and sex). The GWAS was performed on the full imputed dataset of approximately 15,815,000 markers with MAF > 0.01 and info quality score > 0.30. All genomic coordinate positions reported in text are based on build GRCh38.

We filtered association results by P < 1.0 x 10^−4^ and prioritized 379 SNPs (plus 5 internal controls) for replication genotyping on the basis of P-value, minor allele frequency, number of associated traits, number of suggestive SNPs within a locus, size of the locus, gene annotation, and replication assay design score. For the two replication studies, in which we do not have genomewide genotype data from which to derive information on population substructure, we used PLINK [42] linear regression to test genetic association of each of the 227 replication study SNPs that passed quality control filters, testing only the trait with the most significant association in the GWAS for any given replication SNP. We then conducted a meta-analysis of the two replication studies as well as a meta-analysis of the GWAS and the two replication studies using METAL [43]. SNPs were eliminated from downstream meta-analysis due to effect size heterogeneity among the three studies, as indicated by an I^2^ statistic > 50% [44]. Our remaining 24 SNP summary statistics were combined using the inverse variance fixed effects method to determine meta-analysis effect sizes and P-values. A PCA was performed on the 34 phenotype residuals in our unrelated African GWAS sample set to determine the number of effectively independent phenotypes for a Bonferroni correction of our meta-analysis significance threshold. The first 9 eigenvectors had eigenvalues > 1 indicating the variance explained by the first 9 eigenvectors was representative of at least one phenotype and therefore was used in our multiple testing correction. The meta-analysis genomewide significance threshold was calculated using this Bonferroni correction on the GWAS significance threshold for an African population (2.5 x 10^−8^ / 9 = P < 2.78 x 10^−9^) [13]. Conditional analysis were performed by assessing secondary SNP significance before and after the inclusion of the lead SNP as an additional covariate in the model. Regional association plots in **Figs 3**, **4**and **5**were constructed using meta-analysis P-values for the 3 replicated SNPs and GWAS P-values for all other SNPs within the locus of interest using LocusZoom [45].

### Whole Mount *In Situ* Hybridization

Digoxygenin-labeled RNA probes (DIG RNA labeling kit; Roche, Indianapolis, IN) for murine *Pde8a* and *Schip1* were generated by *in vitro* transcription from PCR-derived templates. Wild-type C57BL/6J mice were mated overnight, and the presence of a vaginal plug was taken to indicate embryonic day (E) 0.5. Whole-mount *in situ* hybridization was performed according to standard protocols on mouse embryos harvested from E9.5 to E12.5 time periods and fixed in 4% PFA.

### RNA-seq

For RNAseq analysis, ectoderm and mesenchyme from each paired facial prominence (frontonasal, maxillary, mandibular) of C57BL/6J mice (Jax Labs, Bar Harbor, ME) were collected at E10.5, E11.5 and E12.5 as described previously [46]. RNA was extracted by using Norgen long RNA and microRNA separation kits (Norgen Biotek, Thorold, ON) according to manufacturer’s instructions. RNA-seq libraries were constructed using Illumina TruSeq Stranded mRNA Sample Preparation kits (Illumina, San Diego, CA), and paired-end reads for 125 cycle of sequencing were performed on an Illumina HiSEQ 2500 by the University of Colorado Genomics and Microarray Core. RNA reads were aligned to the mouse genome (MM10) by gSNAP [47], expression (FPKM) derived by Cufflinks [48], and differential expression [49–52] analyzed with ANOVA in R.

## Supporting Information

S1 Fig Age by sex distribution for GWAS, Megacapturor Replication, and Gemini Replication. Age by sex distribution for **(A)** GWAS, *n* = 3,505; **(B)** Megacapturor Replication, *n* = 1,140; and **(C)** Gemini Replication, *n* = 1,250.
(PDF)

S2 Fig Principal components analysis of LD-pruned genomewide markers in unrelated GWAS individuals. Scree plot of the percent of total variance explained by the top 50 PCs **(A)**. PCA cluster plots of the top 4 PCs colored by school **(B)** and tribe **(C)** demonstrate minimal genetic substructure among the top 4 PCs which explain only 0.36% of the total variance.
(PDF)

S3 Fig Distributions of pairwise Fst estimates by school and tribe from LD-pruned markers in unrelated GWAS individuals. Distribution of pairwise Fst estimates by school (**A**) and tribe (**B**) demonstrate minimal genetic differentiation among subgroups.
(PDF)

S4 Fig 3D facial morphs and heat maps of principal components 1–5 (PC1-PC5). Facial morphs and heat maps depict changes associated with positive and negative PC scores for each of the 5 PCs tested for genetic association. See Table 1 for brief descriptions of PC trends.
(PDF)

S5 Fig Midfacial module landmark configuration. Determination of the midfacial module using the RV coefficient. **(A)** The landmarks used in the midfacial module (yellow). **(B)** The total connections among landmarks tested. **(C)** The distribution of the RV coefficient for random subsets of landmarks. The red arrow shows the location of the selected subset from that distribution. **(D)** The shape change that corresponds to the first principal component of the midfacial module.
(PDF)

S6 Fig GWAS Manhattan plots of top signals. GWAS Manhattan plots of **(A)** centroid size, **(B)** PC4, **(C)** and allometry.
(PDF)

S7 Fig *Pde8a* and *Schip1* RNA expression analysis during mouse embryonic facial development. The graphs show RPKM values (reads per kilobase of transcript per million reads mapped) for *Pde8a* (top) and *Schip1* (bottom) derived from RNAseq experiments. Time-course data are shown for the frontonasal prominence (FNP), maxillary prominence (MXP), or mandibular prominence (MNP) at embryonic day (E) 10.5, 11.5 and 12.5 for either the ectoderm (blue) or mesenchyme (red) component. RNAseq experiments were run on independent biological triplicates with error bars showing standard deviation.
(PDF)

S1 File 3D surfaces showing the morphs at the extremes and average points for allometry and PC1-5. (PDF)
