# Supplementary material for: Genomewide Association Study of African Children Identifies Association of SCHIP1 and PDE8A with Facial Size and Shape
Source: PLoS Genet. 2016 Aug 25;12(8):e1006174. doi: 10.1371/journal.pgen.1006174 (PMC4999243; doi:10.1371/journal.pgen.1006174)
Supplement: S1 Fig — Age by sex distribution for (A) GWAS, n = 3,505; (B) Megacapturor Replication, n = 1,140; and (C) Gemini Replication, n = 1,250. (PDF) [file pgen.1006174.s001.pdf]

S1 Fig. Age by sex distribution for GWAS, Megacapturor Replication, and Gemini Replication.

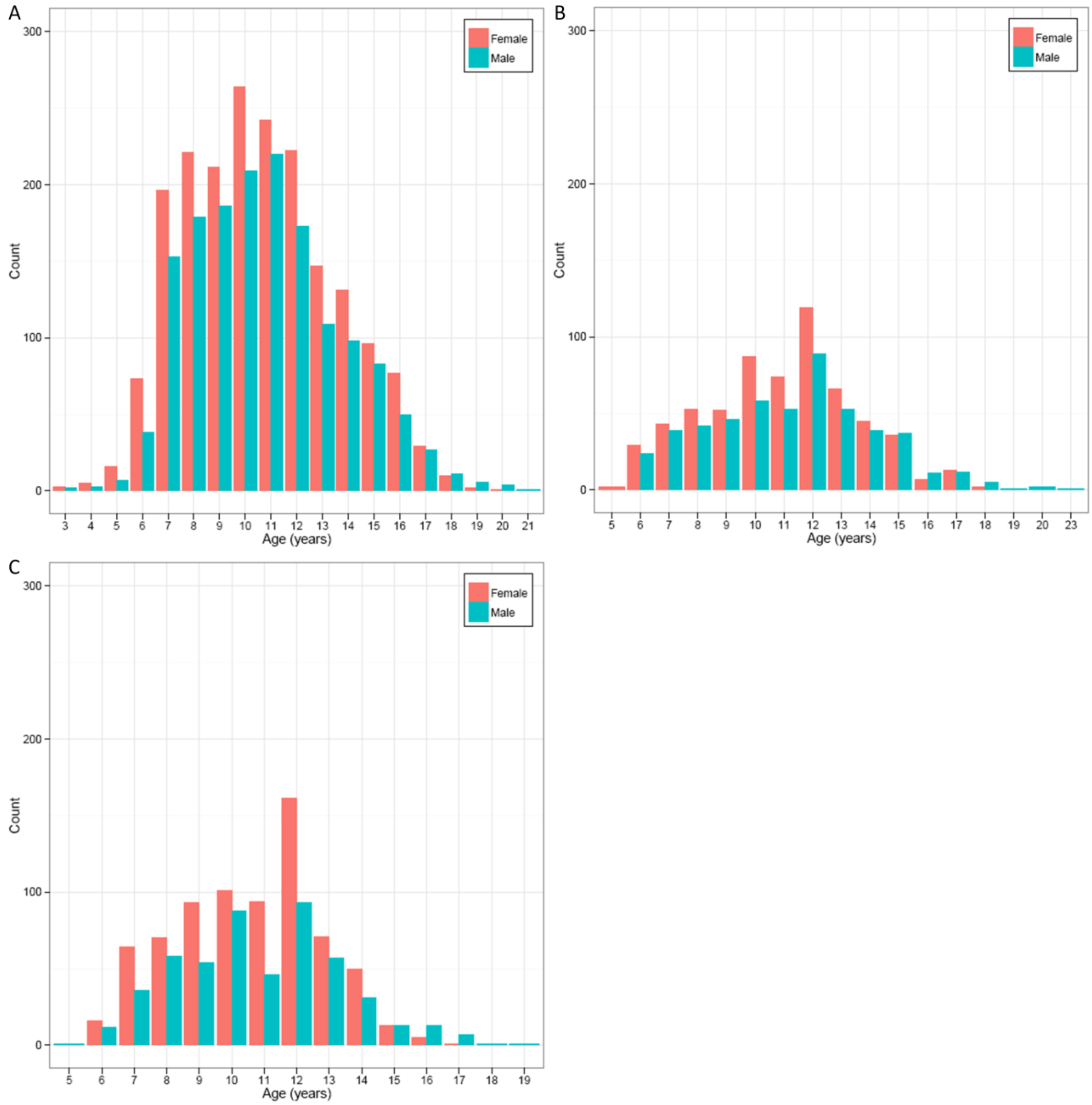

Age by sex distribution for **(A)** GWAS, *n*=3,505; **(B)** Megacapturor Replication, *n*=1,140; and **(C)** Gemini Replication, *n*=1,250.
