# Supplementary material for: Genomewide Association Study of African Children Identifies Association of SCHIP1 and PDE8A with Facial Size and Shape
Source: PLoS Genet. 2016 Aug 25;12(8):e1006174. doi: 10.1371/journal.pgen.1006174 (PMC4999243; doi:10.1371/journal.pgen.1006174)
Supplement: S2 Fig — Scree plot of the percent of total variance explained by the top 50 PCs (A). PCA cluster plots of the top 4 PCs colored by school (B) and tribe (C) demonstrate minimal genetic substructure among the top 4 PCs which explain only 0.36% of the total variance. (PDF) [file pgen.1006174.s002.pdf]

S2 Fig. Principal components analysis of LD-pruned genomewide markers in unrelated GWAS individuals.

A

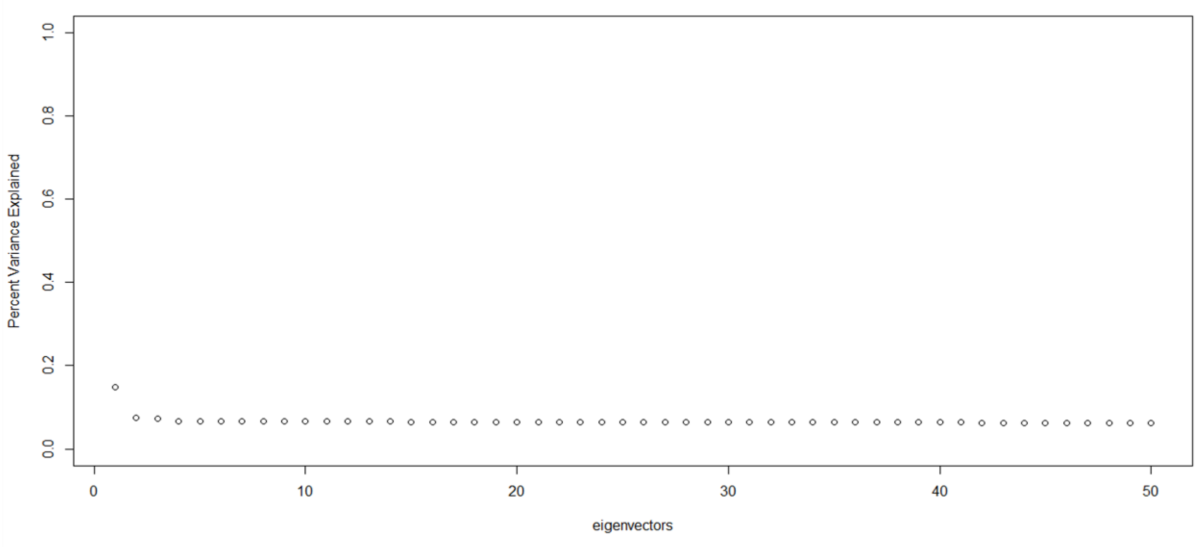

B

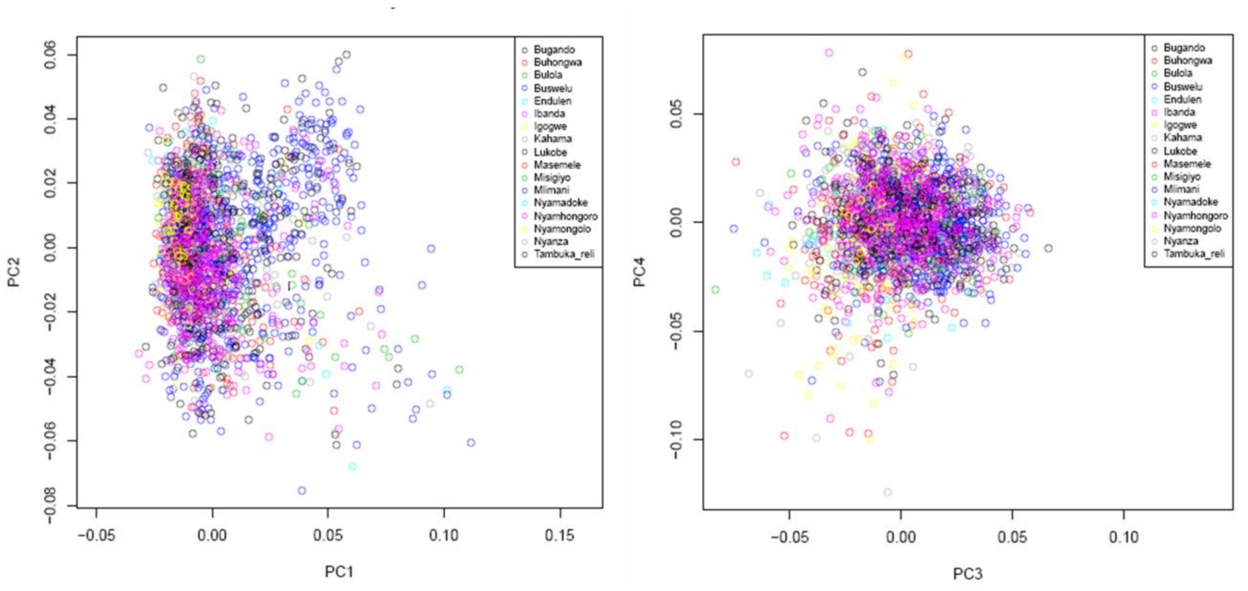

C

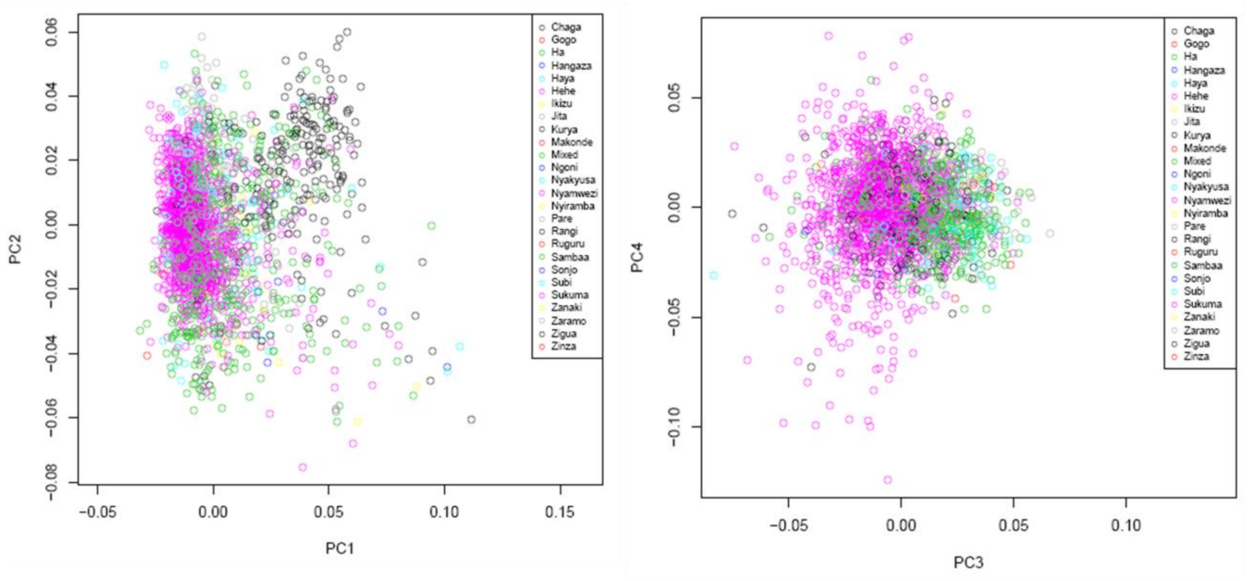

Scree plot of the percent of total variance explained by the top 50 PCs **(A)**. PCA cluster plots of the top 4 PCs colored by school **(B)** and tribe **(C)** demonstrate minimal genetic substructure among the top 4 PCs which explain only 0.36% of the total variance.
