# Supplementary material for: Genomewide Association Study of African Children Identifies Association of SCHIP1 and PDE8A with Facial Size and Shape
Source: PLoS Genet. 2016 Aug 25;12(8):e1006174. doi: 10.1371/journal.pgen.1006174 (PMC4999243; doi:10.1371/journal.pgen.1006174)
Supplement: S3 Fig — Distribution of pairwise Fst estimates by school (A) and tribe (B) demonstrate minimal genetic differentiation among subgroups. (PDF) [file pgen.1006174.s003.pdf]

S3 Fig. Distributions of pairwise Fst estimates by school and tribe from LD-pruned markers in unrelated GWAS individuals.

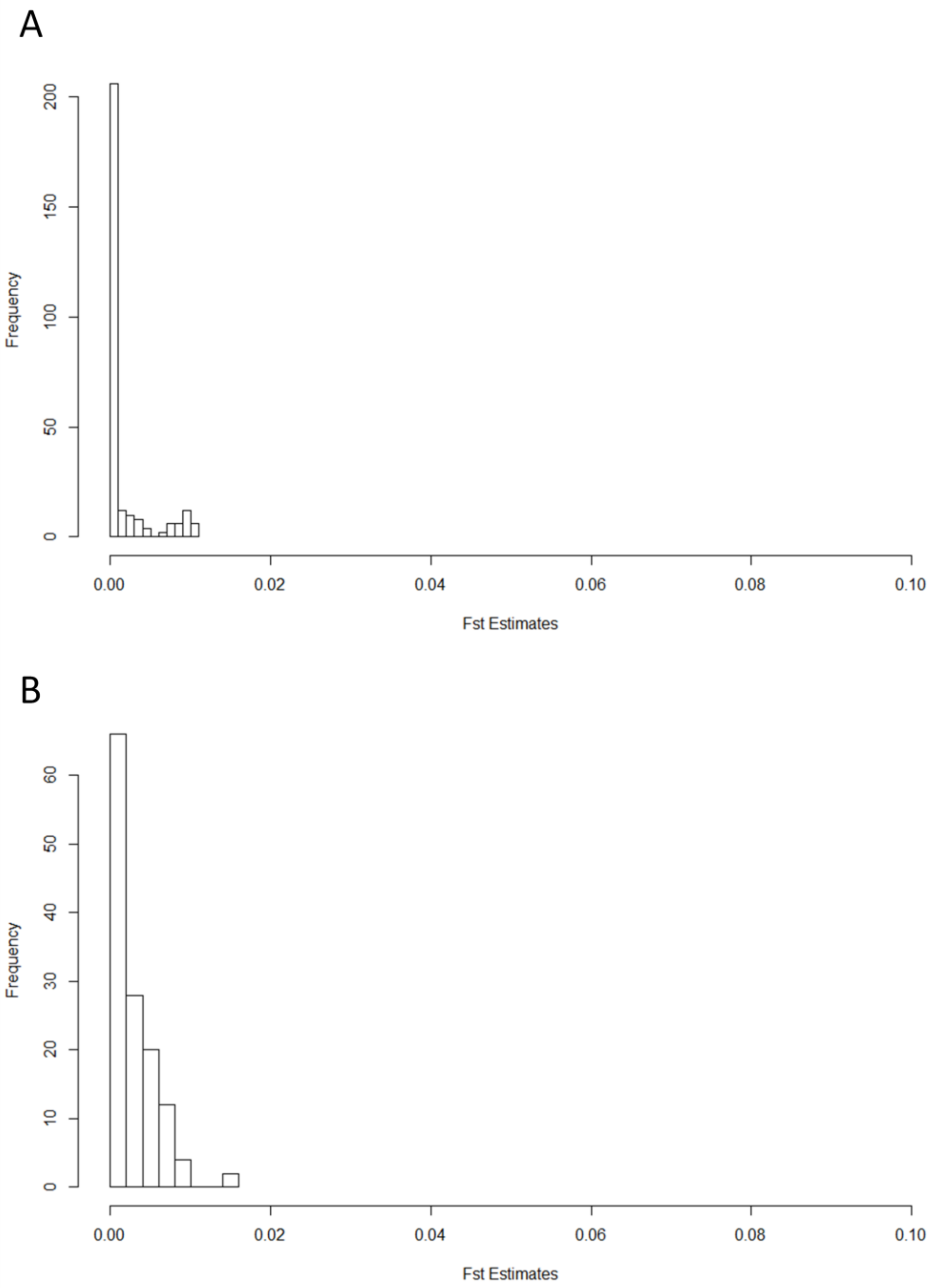

Distribution of pairwise Fst estimates by school (**A**) and tribe (**B**) demonstrate minimal genetic differentiation among subgroups.
