# Supplementary material for: Genomewide Association Study of African Children Identifies Association of SCHIP1 and PDE8A with Facial Size and Shape
Source: PLoS Genet. 2016 Aug 25;12(8):e1006174. doi: 10.1371/journal.pgen.1006174 (PMC4999243; doi:10.1371/journal.pgen.1006174)
Supplement: S4 Fig — Facial morphs and heat maps depict changes associated with positive and negative PC scores for each of the 5 PCs tested for genetic association. See Table 1 for brief descriptions of PC trends. (PDF) [file pgen.1006174.s004.pdf]

S4 Fig. 3D facial morphs and heat maps of principal components 1-5 (PC1-PC5).

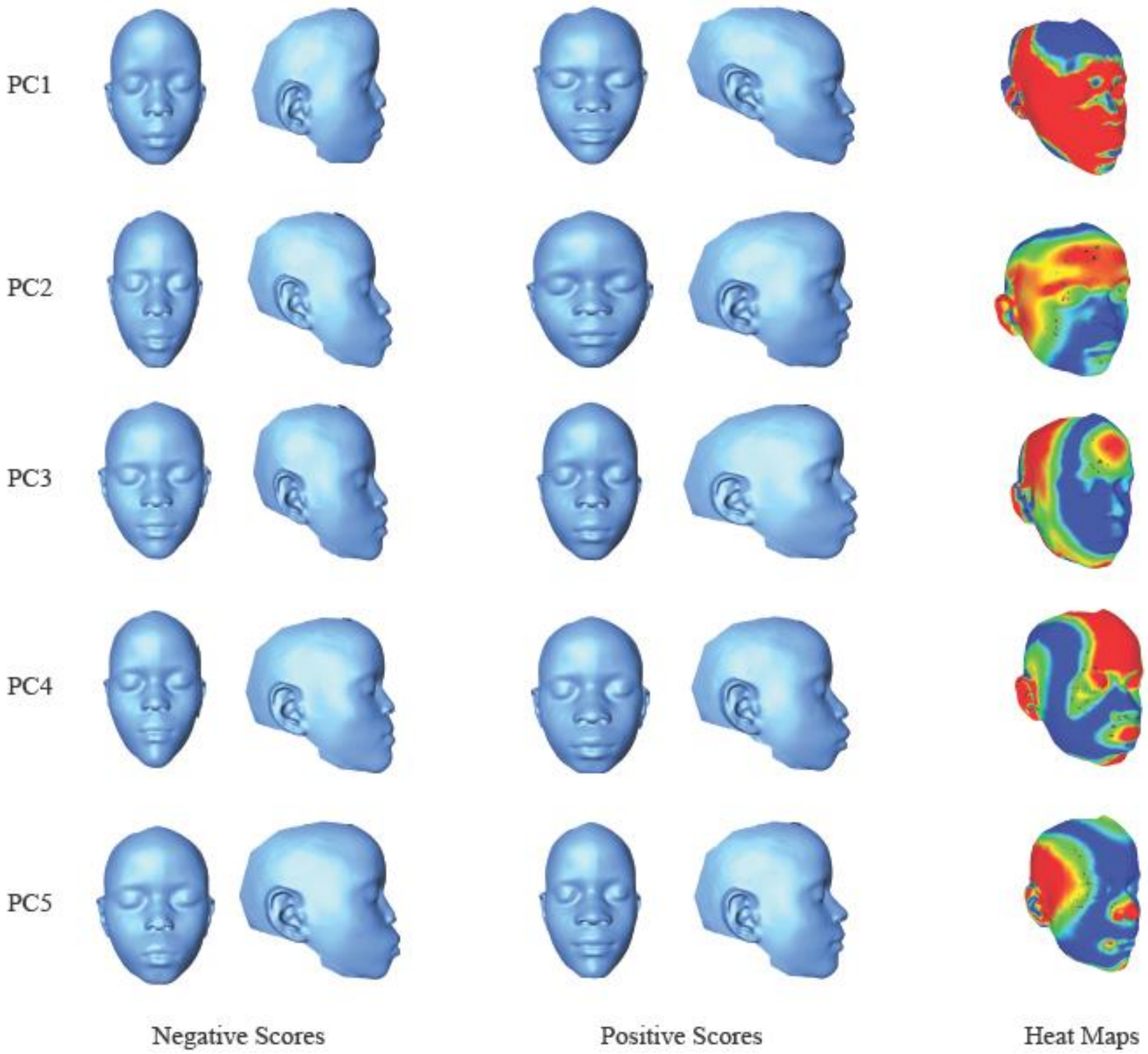

Facial morphs and heat maps depict changes associated with positive and negative PC scores for each of the 5 PCs tested for genetic association. See Table 1 for brief descriptions of PC trends.
