# Supplementary material for: Genomewide Association Study of African Children Identifies Association of SCHIP1 and PDE8A with Facial Size and Shape
Source: PLoS Genet. 2016 Aug 25;12(8):e1006174. doi: 10.1371/journal.pgen.1006174 (PMC4999243; doi:10.1371/journal.pgen.1006174)
Supplement: S5 Fig — Determination of the midfacial module using the RV coefficient. (A) The landmarks used in the midfacial module (yellow). (B) The total connections among landmarks tested. (C) The distribution of the RV coefficient for random subsets of landmarks. The red arrow shows the location of the selected subset from that distribution. (D) The shape change that corresponds to the first principal component of the midfacial module. (PDF) [file pgen.1006174.s005.pdf]

S5 Fig. Midfacial module landmark configuration.

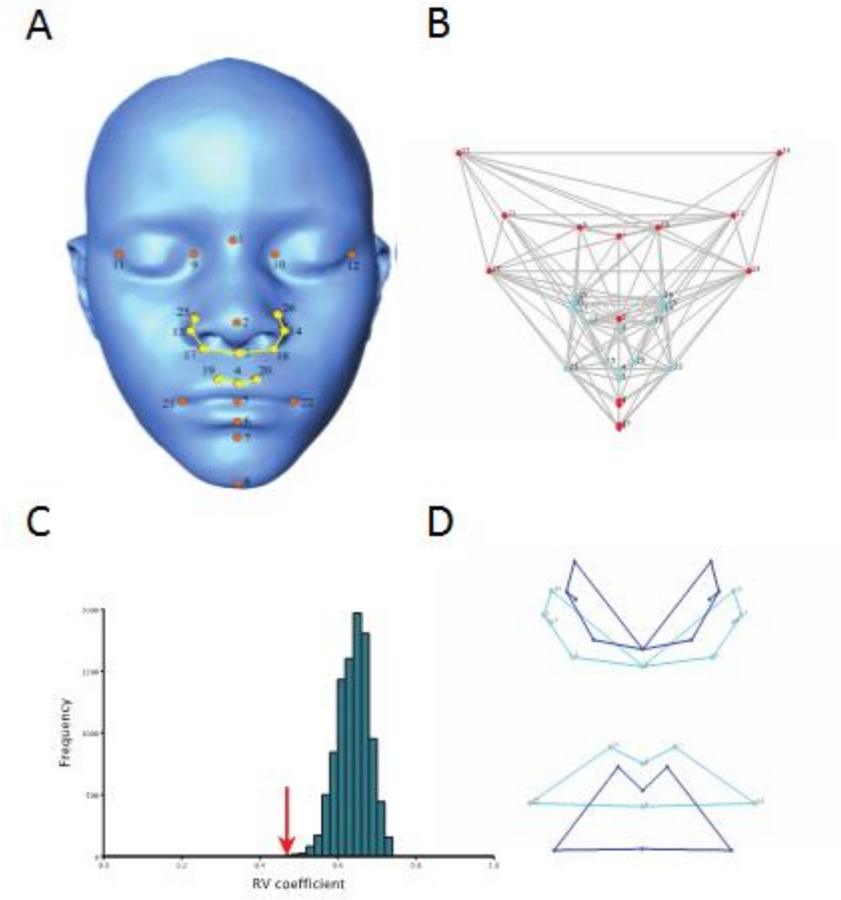

Determination of the midfacial module using the RV coefficient[1]. **(A)** The landmarks used in the midfacial module (yellow). **(B)** The total connections among landmarks tested. **(C)** The distribution of the RV coefficient for random subsets of landmarks. The red arrow shows the location of the selected subset from that distribution. **(D)** The shape change that corresponds to the first principal component of the midfacial module.

1. Klingenberg, C.P. Morphometric integration and modularity in configurations of landmarks: Tools for evaluating a priori hypotheses. *Evol. Dev.* **11**, 405-421 (2009).
