# Supplementary material for: Genomewide Association Study of African Children Identifies Association of SCHIP1 and PDE8A with Facial Size and Shape
Source: PLoS Genet. 2016 Aug 25;12(8):e1006174. doi: 10.1371/journal.pgen.1006174 (PMC4999243; doi:10.1371/journal.pgen.1006174)
Supplement: S6 Fig — GWAS Manhattan plots of (A) centroid size, (B) PC4, (C) and allometry. (PDF) [file pgen.1006174.s006.pdf]

S6 Fig. GWAS Manhattan plots of top signals.

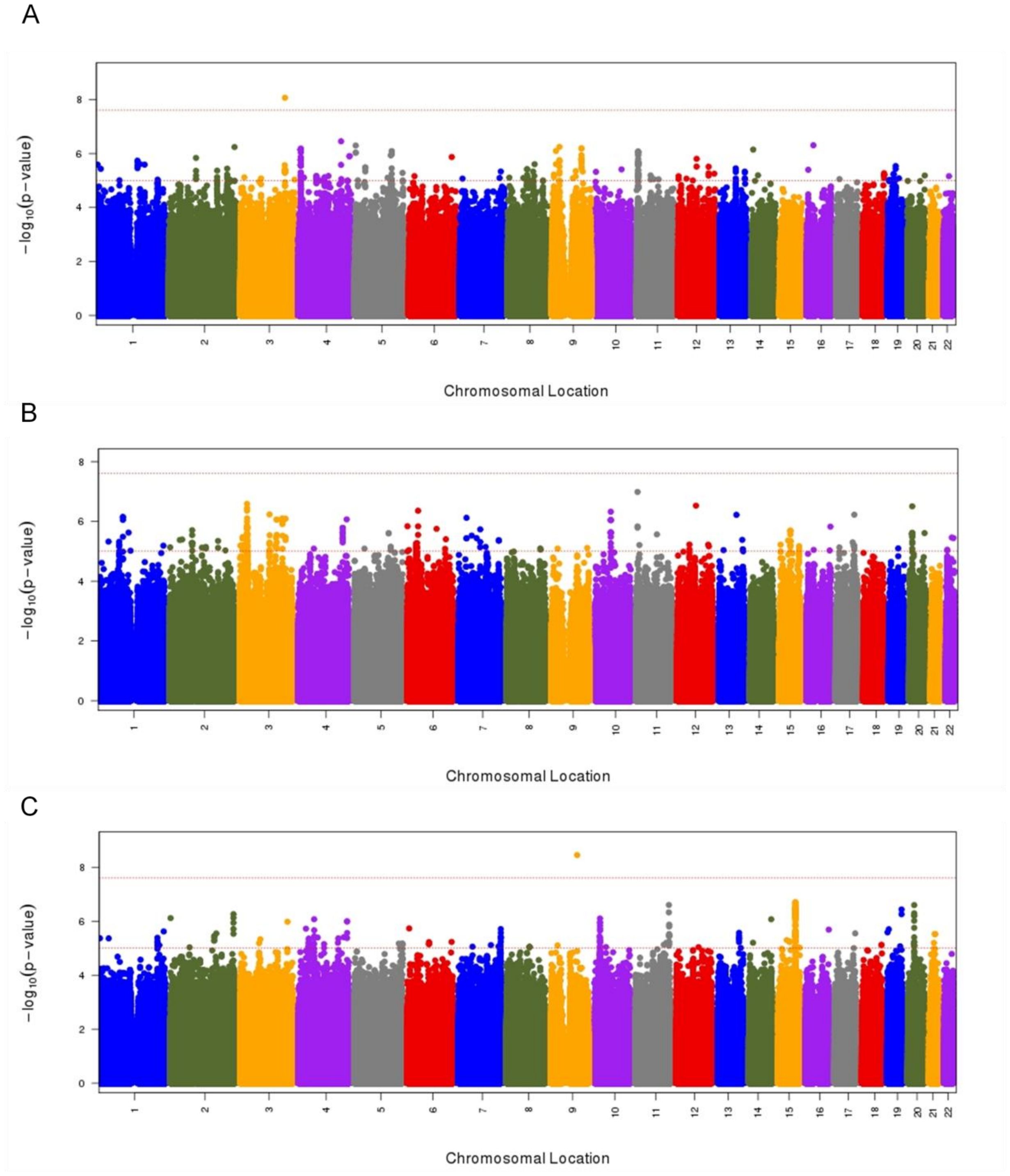

GWAS Manhattan plots of **(A)** centroid size, **(B)** PC4, **(C)** and allometry.
