# Supplementary material for: Genomewide Association Study of African Children Identifies Association of SCHIP1 and PDE8A with Facial Size and Shape
Source: PLoS Genet. 2016 Aug 25;12(8):e1006174. doi: 10.1371/journal.pgen.1006174 (PMC4999243; doi:10.1371/journal.pgen.1006174)
Supplement: S7 Fig — The graphs show RPKM values (reads per kilobase of transcript per million reads mapped) for Pde8a (top) and Schip1 (bottom) derived from RNAseq experiments. Time-course data are shown for the frontonasal prominence (FNP), maxillary prominence (MXP), or mandibular prominence (MNP) at embryonic day (E) 10.5, 11.5 and 12.5 for either the ectoderm (blue) or mesenchyme (red) component. RNAseq experiments were run on independent biological triplicates with error bars showing standard deviation. (PDF) [file pgen.1006174.s007.pdf]

S7 Fig. *Pde8a* and *Schip1* RNA expression analysis during mouse embryonic facial development.

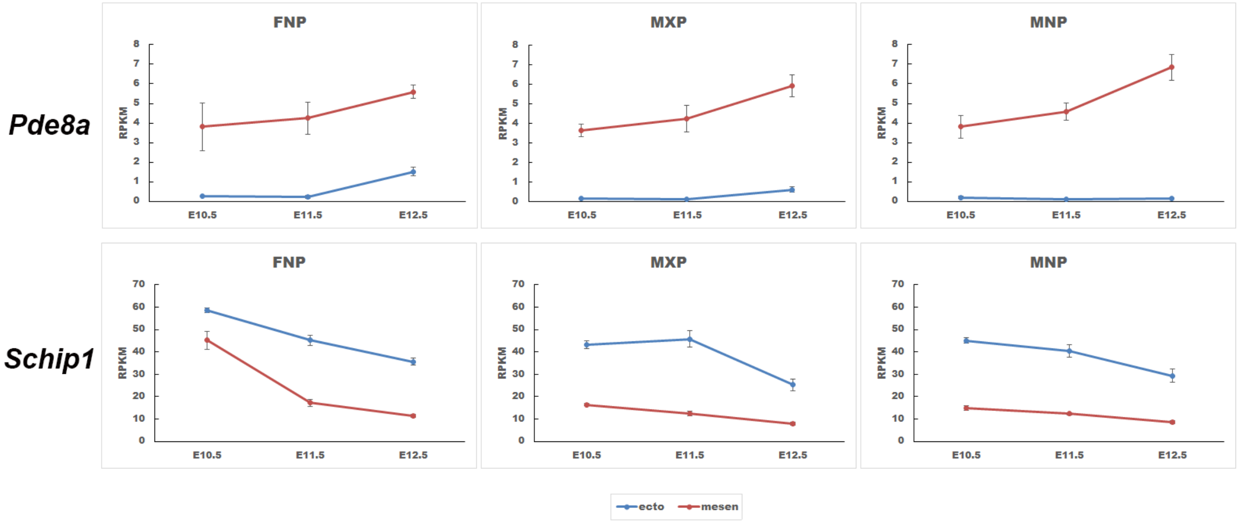

The graphs show RPKM values (reads per kilobase of transcript per million reads mapped) for *Pde8a* (top) and *Schip1* (bottom) derived from RNAseq experiments. Time-course data are shown for the frontonasal prominence (FNP), maxillary prominence (MXP), or mandibular prominence (MNP) at embryonic day (E) 10.5, 11.5 and 12.5 for either the ectoderm (blue) or mesenchyme (red) component. RNAseq experiments were run on independent biological triplicates with error bars showing standard deviation.
