# Supplementary material for: Genomewide Association Study of African Children Identifies Association of SCHIP1 and PDE8A with Facial Size and Shape
Source: PLoS Genet. 2016 Aug 25;12(8):e1006174. doi: 10.1371/journal.pgen.1006174 (PMC4999243; doi:10.1371/journal.pgen.1006174)
Supplement: S1 File — (PDF) [file pgen.1006174.s008.pdf]

S1 File. 3D surfaces showing the morphs at the extremes and average points for allometry and PC1-5. For each image, click on it to move it to the frontal view. The faces an then be rotated by holding the mouse or trackpad key down or by touch on a touchscreen. The morphs are scaled so as to represent the observed extremes of each distribution.

Click on each image to place in default view.

Allometry

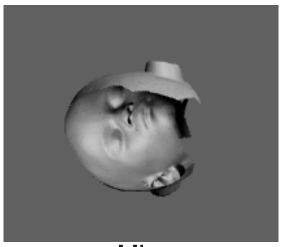
Min

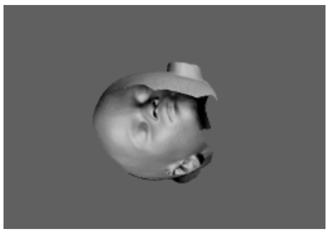
Average

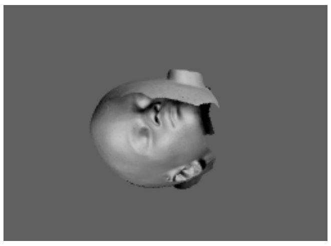
Max

PC1

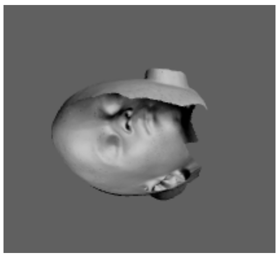
Min

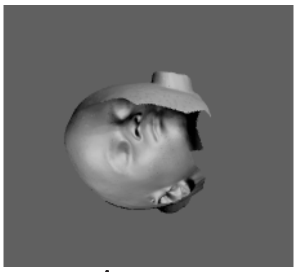
Average

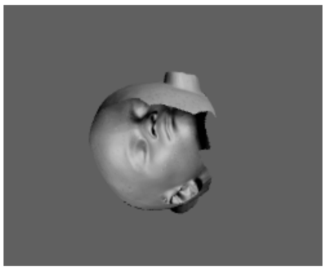
Max

PC2

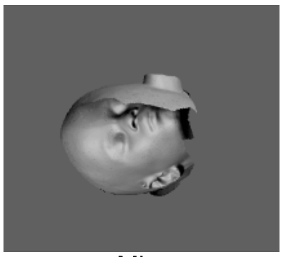
Min

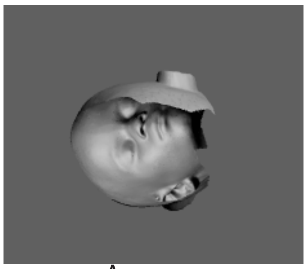
Average

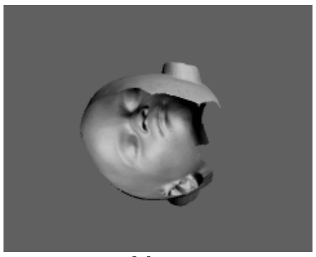
Max

PC3

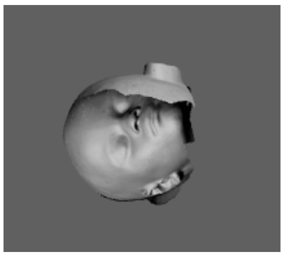
Min

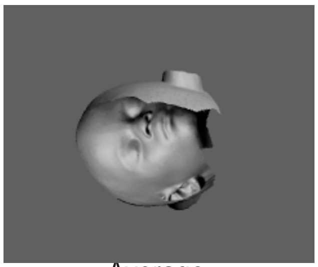
Average

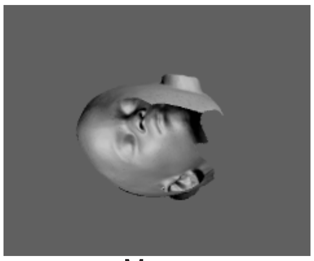
Max

PC4

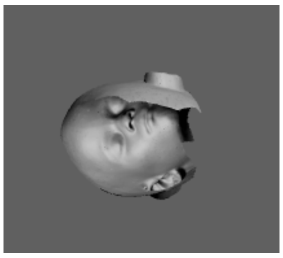
Min

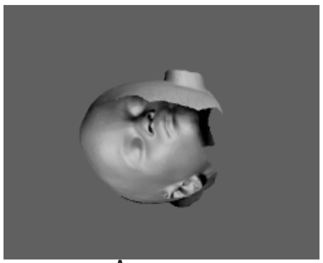
Average

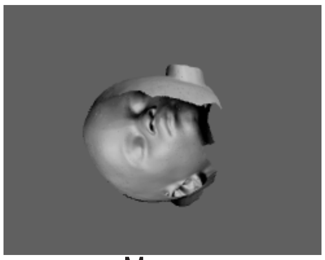
Max

PC5

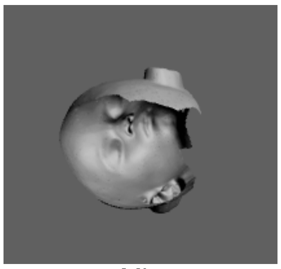
Min

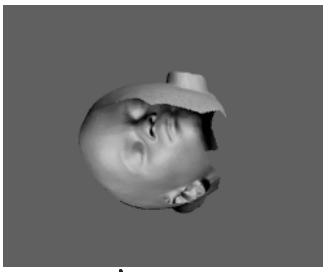
Average

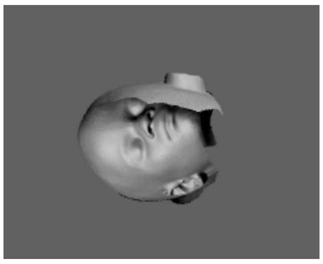
Max
